# Regulation of proton partitioning in kinase-activating acute myeloid leukemia and its therapeutic implication

**DOI:** 10.1038/s41375-022-01606-0

**Published:** 2022-05-27

**Authors:** Cheuk-Him Man, Xiaoyuan Zeng, Wing Lam, Timothy C. C. Ng, Tsz-Ho Kwok, Kenny C. C. Dang, Thomas W. Y. Leung, Nelson K. L. Ng, Stephen S. Y. Lam, Chae-Yin Cher, Anskar Y. H. Leung

**Affiliations:** 1 Division of Haematology, Department of Medicine, Li Ka Shing Faculty of Medicine, the University of Hong Kong, Hong Kong SAR, China; 2 Centre for Oncology and Immunology, Hong Kong Science Park, Hong Kong SAR, China

**Keywords:** Acute myeloid leukaemia, Translational research

## Abstract

Gain-of-function kinase mutations are common in AML and usually portend an inferior prognosis. We reported a novel mechanism whereby kinase mutants induced intracellular alkalization characteristic in oncogenesis. Thirteen kinases were found to activate sodium/hydrogen exchanger (NHE1) in normal hematopoietic progenitors, of which FLT3-ITD, KRAS^G12D^, and BTK phosphorylated NHE1 maintained alkaline intracellular pH (pHi) and supported survival of AML cells. Primary AML samples with kinase mutations also showed increased NHE1 phosphorylation and evidence of NHE1 addiction. Amiloride enhanced anti-leukemic effects and intracellular distribution of kinase inhibitors and chemotherapy. Co-inhibition of NHE1 and kinase synergistically acidified pHi in leukemia and inhibited its growth in vivo. Plasma from patients taking amiloride for diuresis reduced pHi of leukemia and enhanced cytotoxic effects of kinase inhibitors and chemotherapy in vitro. NHE1-mediated intracellular alkalization played a key pathogenetic role in transmitting the proliferative signal from mutated-kinase and could be exploited for therapeutic intervention in AML.

## Introduction

Acute myeloid leukemia (AML) is a group of heterogeneous diseases with distinct clinicopathologic, cytogenetic, and genetic characteristics, sharing in common an abnormal increase of myeloblasts in blood and bone marrow (BM). Induction chemotherapy and allogeneic hematopoietic stem cell transplantation are the mainstays of treatment, with a cure rate of 30–40% in eligible patients [1]. Recently, targeted therapies for AML have been approved for specific AML subtypes and clinical settings [2] often based on improvement, albeit modest, in overall survivals. However, the overall outcome of this disease has remained unsatisfactory and in patients who are ineligible for these treatments, the prognosis is dismal.

Gain-of-function tyrosine kinase mutations in AML often portend an inferior prognosis when treated with conventional chemotherapy. For instance, internal tandem duplication of FMS-Like Tyrosine Kinase 3 (*FLT3*-ITD) is one of the most common mutations in cytogenetically normal (CN) AML and confers adverse outcomes [3]. FLT3 inhibitor, midostaurin, was approved for use in combination with induction and consolidation chemotherapy [4]. However, relapse is a common occurrence, limiting its clinical benefits. *RAS* mutations are also common but effective inhibitor is unavailable. *KIT* mutation occurs in nearly 50% of core-binding factor AML and confers an inferior outcome to this hitherto favorable AML subtype [5]. Tyrosine kinase inhibitors targeting *KIT* mutation in combination with chemotherapy are being tested in Phase III clinical trials and the benefits remain to be determined [6]. Novel therapeutic strategies are much needed for kinase-mutated AML subtypes.

A high intracellular pH (pHi) has been shown to play a critical role in oncogenesis with diverse pathogenetic sequelae [7, 8]. Lowering pHi by as little as 0.1–0.2 pH unit could perturb these pathologic processes and might be harnessed for therapeutic intervention. A number of regulators have been shown to regulate pHi during oncogenesis, including monocarboxylate transporters (MCT) [9–11]; Na^+^/H^+^ exchangers (NHE) [7, 12]; vacuolar ATPase (V-ATPase) [13]; Cl^−^/HCO3^−^ anion exchangers (AE) [14]; Na^+^/HCO3^−^ co-transporters [15]; and carbonic anhydrases (CA) [16, 17]. However, clinically applicable therapeutic agents targeting these pHi regulators are presently lacking. An exception is amiloride, a safe and effective NHE1 inhibitor that has been used for decades as a diuretic agent.

NHE1 comprises an N-terminal transmembrane transport domain and a C-terminal cytoplasmic regulatory domain. The latter provides the structural anchor between cytoskeletal proteins and plasma membrane as well as the docking and phosphorylation sites for regulatory signals. Under physiological conditions, NHE1 maintains pHi by H^+^ efflux in exchange for Na^+^ and is activated when pHi falls below the set-point. Oncogenic kinases may induce aberrant NHE1 activation by protein docking and C-terminal phosphorylation, shifting its set-point to favor intracellular alkalinization [18, 19]. We hypothesized that NHE1 may be activated in kinase-mutated AML and the resulting intracellular alkalinization may play a role in leukemogenesis. Integration of NHE1 inhibitor in the treatment algorithm of AML may improve treatment efficacy and outcome for these diseases.

## Results

### Activation of NHE1 by kinase expression

**Fig. 1:**
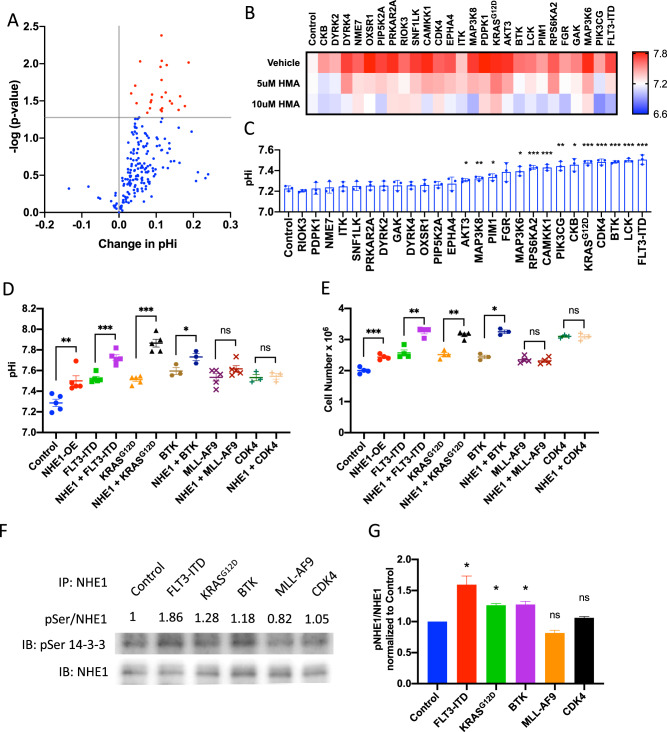
Kinase activation is crucial in enhancing NHE1 activity in AML. **A** Volcano plot of kinase overexpression (–log(*p* value) against change in pHi in HEK293, each dot represents one kinase, red dots indicated those kinases that significantly raised the pHi (*n* = 3). **B** Heatmap showing pHi of HEK293 that increased with kinase overexpression and was reduced by HMA treatment in vitro (*n* = 3). **C** Thirteen kinases raised pHi of cord blood progenitors in vitro (*n* = 3). **D**, **E** NHE1 overexpression enhanced **D** pHi increase and **E** growth of cord blood progenitors upon leukemic alleles or kinases overexpression in vitro (FLT3-ITD, KRAS^G12D^, and BTK), but not with MLL-AF9 or CDK4 (*n* = 3–5). **F** Representative western blot analysis of phosphoserine 14-3-3 (pSer 14-3-3) and NHE1 in NHE1 immunoprecipitation (IP) eluate from cord blood progenitors upon leukemic alleles or kinase overexpression. The numbers representing the level of NHE1 phosphorylation (intensity of pSer 14-3-3 signal normalized by NHE1 in NHE1 IP eluate) are summarized in panel **G**. **G** FLT3-ITD, KRAS^G12D^, and BTK, but not MLL-AF9 or CDK4 increased the level of NHE1 phosphorylation in cord blood progenitors (*n* = 3).

A kinase screen was performed to identify upstream signals of NHE1 [20] using a fluorescent pH reporter [21]. A library comprising 195 human kinases and kinase-related open reading frames was over-expressed in HEK293-mChSE (mCherry-SEpHluorin) (Fig. 1A). Overexpression of 26 kinases was found to induce a significant rise in pHi of HEK293 cells (Fig. 1B), which was reversible by a specific NHE1 inhibitor HMA [5-(N,N-hexamethylene) amiloride)], attesting to the dominant role of NHE1 in pH regulation by these kinases. Thirteen of them induced a significant rise in pHi in human cord blood (CB) CD34^+^ progenitors (Fig. 1C). To ascertain the roles of kinase activation in NHE1 function, selected kinases were co-transduced with *NHE1* into CB CD34^+^ progenitor cells. Expression of *FLT3*-ITD, *KRAS*^G12D^, and *BTK*, but not cyclin-dependent kinase 4 (*CDK4*) or *MLL*-*AF9* that mediates leukemogenesis via epigenetic remodeling [22, 23], induced intracellular alkalinization and promoted cell growth in the presence of *NHE1* expression (Fig. 1D, E). Increased phosphorylation of NHE1 protein could be demonstrated by overexpression of *FLT3*-ITD, *KRAS*^G12D^, and *BTK*, but not *CDK4* or *MLL-AF9* (Fig. 1F, G).

### Therapeutic inhibition of NHE1 in AML

**Fig. 2:**
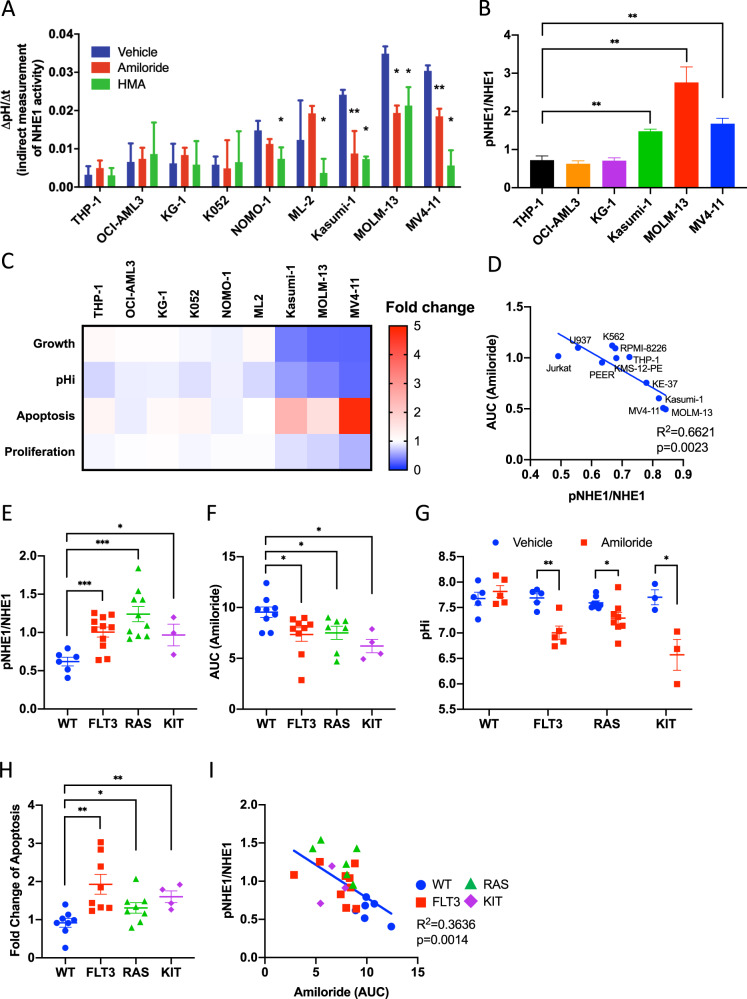
Sensitivity toward therapeutic NHE1 inhibition in different AML subtypes. **A** Kasumi-1, MOLM-13, and MV4-11, but not other human AML cell lines, showed higher NHE1 activity and were sensitive to amiloride (10 μM) and HMA (10 μM) (*n* = 5). **B** Kasumi-1, MOLM-13, and MV4-11 (amiloride-sensitive AML) showed higher level of NHE1 phosphorylation compared to THP-1, OCI-AML3, and KG-1 (amiloride-resistant AML) (*n* = 3) (see also Supplementary Fig. S1A for the western blot analysis). **C** Heatmap summarizing the effects (fold changes of growth, pHi, apoptosis, and proliferation normalized to vehicle control) of in vitro amiloride treatment on different human AML cell lines (*n* = 4) (see Supplementary Fig. S1B–E for the raw data to the heatmap). **D** Level of NHE1 phosphorylation significantly correlated with the area-under-curve (AUC) of amiloride treatment in different human leukemic cell lines (*n* = 10). **E**–**H** Primary AML samples with *FLT3*, *RAS,* or *KIT* mutation showed **E** higher level of NHE1 phosphorylation (*n* = 30), **F** increased sensitivity toward in vitro amiloride treatment (*n* = 29), **G** intracellular acidification upon in vitro amiloride treatment (*n* = 21), and **H** increased apoptosis induction upon in vitro amiloride treatment (*n* = 21), compared to WT samples. **I** Level of NHE1 phosphorylation significantly correlated with the AUC of amiloride treatment in primary AML samples (*n* = 25). Amiloride concentration was 10 μM in these experiments.

The observations aforementioned supported the proposition that kinase-driven AML may be addicted to NHE1 phosphorylation and intracellular alkalinization and hence may be sensitive to NHE1 inhibition. To prove the principle, nine AML cell lines (tested mycoplasma free), representing diverse driver events in leukemogenesis, from DSMZ or ATCC (Supplementary Table S1), were tested for their sensitivity to HMA and amiloride. Suppression of NHE1 activity by these agents, as evaluated by ΔpH/Δt, supported the proposition that NHE1 is operative. Importantly, Kasumi-1, MOLM-13, and MV4-11, characterized by activating *KIT* mutation and *FLT3*-ITD, showed significantly higher NHE1 activity (Fig. 2A) and higher level of phospho-NHE1 (Fig. 2B and Supplementary Fig. S1A) that was susceptible to NHE1 inhibition. Furthermore, these three cell lines were significantly more sensitive to amiloride (Fig. 2C) in decreasing cell number, intracellular acidification, induction of apoptosis, and suppression of cellular proliferation compared to vehicle control. The heatmap showed the effects of amiloride on the growth, pHi, apoptosis, and proliferation in different cell lines, with the fold change of these parameters (relative to vehicle control) being represented by colors as encoded in the sidebar. The raw data were shown in Supplementary Fig. S1B–E, where the effects of HMA were also presented. Sensitivity toward amiloride and NHE1 phosphorylation were significantly correlated in other hematologic malignancies (Fig. 2D). To test if these observations were clinically relevant, we examined the sensitivity of primary human AML samples carrying *FLT3*-ITD, *KIT,* or *RAS* mutations to amiloride treatment. These samples showed increased NHE1 phosphorylation compared to triple-negative cases (*FLT3*-WT, *KIT*-WT, and *RAS*-WT) (Fig. 2E and Supplementary Fig. S1F) and were more sensitive to amiloride in growth suppression, intracellular acidification, and apoptosis induction (Fig. 2F–H). Similar to AML cell lines, sensitivity to amiloride in primary AML samples was significantly correlated with their NHE1 phosphorylation (Fig. 2I). Collectively, the results showed that activating kinase mutations in AML were associated with NHE1 phosphorylation and sensitivity to NHE1 inhibition.

### Knockdown of NHE1 in different AML

**Fig. 3:**
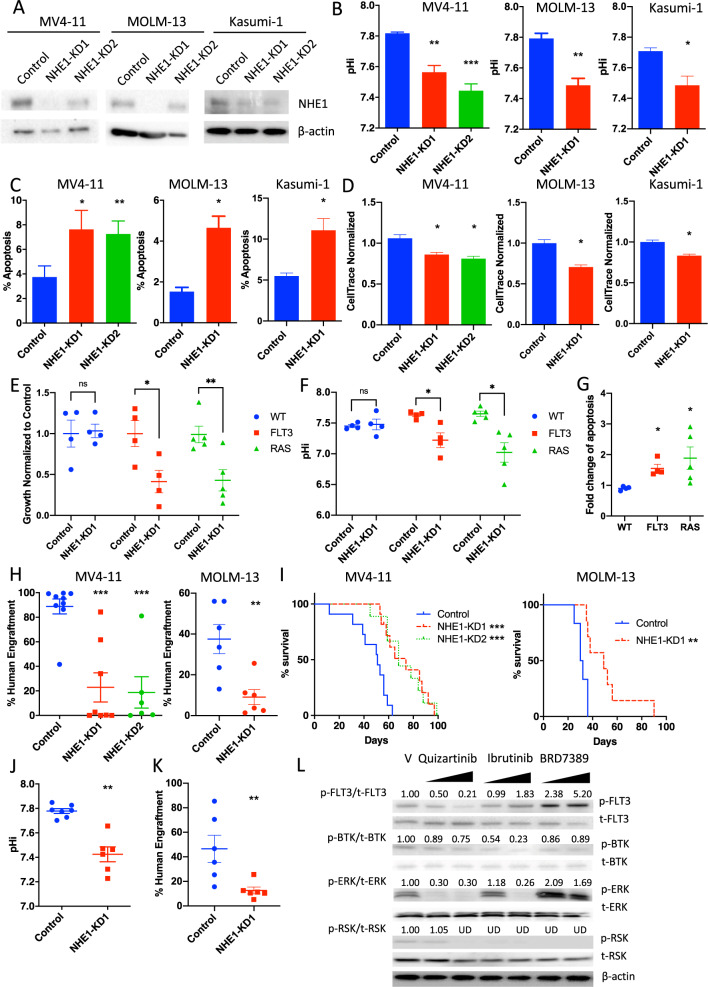
Knockdown of NHE1 suppressed the growth of kinase-mutated AML. **A** Representative western blot analysis showing successful *NHE1* knockdown by shRNA in MV4-11, MOLM-13, and Kasumi-1. **B**–**D** In vitro *NHE1* knockdown **B** reduced pHi, **C** induced apoptosis, and **D** suppressed proliferation in MV4-11, MOLM-13, and Kasumi-1 compared to scrambled shRNA control (*n* = 3). **E**–**G** In vitro *NHE1* knockdown **E** suppressed the growth, **F** reduced pHi, and **G** induced apoptosis (normalized to scrambled shRNA control) in primary AML samples carrying *FLT3* or *RAS* mutation, but not in AML with wildtype *FLT3* or *RAS* (*n* = 13). **H** *NHE1*-KD suppressed the human leukemia engraftment of MV4-11 and MOLM-13 in NSG mice, compared to scrambled shRNA control, at week 5 and week 3 post transplantation respectively (*n* = 6–9). **I** Survival analysis of NSG mice engrafted with MV4-11 and MOLM-13 carrying *NHE1*-KD showed longer survival compared to those with scrambled shRNA control (*n* = 7–11). **J** *NHE1*-KD reduced ex vivo pHi MV4-11 engrafting in NSG compared to scrambled shRNA control (*n* = 6–7). **K** *NHE1*-KD suppressed human leukemia engraftment of primary AML samples with *FLT3* or *RAS* mutation in NSG mice, compared to scrambled shRNA control, at week 12–16 post transplantation (*n* = 6). **L** Representative western blot analysis of kinase activation upon therapeutic inhibition in MV4-11 (quizartinib: 10–100 nM; ibrutinib: 5–10 μM; BRD7389: 5–10 μM) (the number showing the ratio of phosphorylated kinase to the corresponding total kinase normalized by the vehicle control. UD undetected).

To confirm the pathogenetic role of NHE1 in amiloride-sensitive AML, *NHE1* was knocked down (KD) by shRNAs in MV4-11, MOLM-13, and Kasumi-1 (Fig. 3A). OCI-AML3 and THP-1 were used as amiloride-resistant control cell lines. Compared with scrambled shRNA control, *NHE1*-KD significantly lowered pHi (Fig. 3B), induced apoptosis (Fig. 3C), and suppressed proliferation (Fig. 3D) in amiloride-sensitive but not in resistant cell lines (Supplementary Fig. S2A–C). *NHE1*-KD in primary AML with *FLT3*-ITD and *RAS* mutations (Supplementary Fig. S2D) significantly suppressed in vitro growth (Fig. 3E), lowered pHi (Fig. 3F), and induced apoptosis (Fig. 3G) compared with samples without these mutations. *NHE1*-KD reduced MV4-11 and MOLM-13 engraftment in the xenotransplantation model and prolonged animal survival (Fig. 3H, I). Engrafting leukemia cells with *NHE1*-KD had a lower pHi at 6 weeks post transplantation (Fig. 3J) suggesting effective suppression of NHE1 activity and its association with reduced growth in vivo. In vivo effect of *NHE1*-KD was further confirmed in kinase-mutated primary AML (Fig. 3K, FLT3: *n* = 4, RAS: *n* = 2).

### Upstream activation of NHE1 by kinases in AML

To identify mechanisms of upstream activations of NHE1, NHE1 active MV4-11 and Kasumi-1 were treated with specific inhibitors to kinases (FLT3-ITD/KIT, BTK, CDK4), whose effects on intracellular alkalization were among the most significant in CB. p90-RSK, a known activator of NHE1, was included as a positive control [18]. In MV4-11, quizartinib (FLT3-ITD/KIT inhibitor), ibrutinib (BTK inhibitor), and BRD7389 (p90-RSK inhibitor) but not ribociclib (CDK4/6 inhibitor) significantly reduced phosphorylation of NHE1 (Supplementary Fig. S3A). All four inhibitors significantly reduced pHi (Supplementary Fig. S3B). In Kasumi-1, quizartinib and ibrutinib but not ribociclib, significantly reduced NHE1 phosphorylation (Supplementary Fig. S3C). All of them significantly reduced pHi (Supplementary Fig. S3D). Inhibition of calmodulin (CGS9343B) and ROCK-RhoA (HA1100), which have been shown to regulate NHE1 activity in cardiac myocytes [24] and fibroblast cells [25], had no effect on cell growth or pHi in AML cell lines (Supplementary Fig. S3E, F).

### Kinase-specific phosphorylation of NHE1

To investigate the molecular mechanisms of NHE1 phosphorylation in AML, site-directed mutagenesis was performed at putative kinase substrate sites in its cytoplasmic tail (amino acids 648-711) and the effects of kinase inhibitors were examined. The phosphorylation sites of NHE1 and the corresponding kinase have been reported, in particular S648 by AKT [19], S703 by p90-RSK [26], S766/S770/S771 (SSS) by ERK [27], and T718/S723/S726/S729 (TSSS) by p38 MAPK [28]. Mutations of serine/threonine to alanine at S648A, S703A and simultaneous mutations at S766A, S770A, and S771A (SSSA) diminished the effects of ibrutinib whereas these mutations and those at T718, S723, S726, and S729 (TSSSA) diminished the effects of quizartinib (Supplementary Fig. S3G). Mutations at S703A and SSS, but not other sites, abolished intracellular acidification by ravoxertinib, an ERK1/2 inhibitor, which is consistent with ERK/p90-RSK axis in NHE1 activation [18]. None of these mutations affected pHi response to ribociclib. The results suggested that phosphorylation of NHE1 at specific sites mediated the pathogenic effects of BTK and FLT3-ITD in AML.

To demonstrate a direct interaction between activating kinases and NHE1, co-immunoprecipitation was performed in MV4-11, characterized by *FLT3*-ITD, the most common activating kinase mutation in AML. Co-immunoprecipitation was observed between NHE1 and kinases, such as FLT3, BTK, and ERK (Supplementary Fig. S3H). The results indicated that, in addition to their downstream effector ERK/p90-RSK [18], FLT3 and BTK might activate NHE1 by direct binding and phosphorylation (Fig. 3L). To ascertain the functional link between FLT3 and NHE1 activity, we made use of the differential effects of FLT3 inhibitors on FLT3-ITD and FLT3-ITD+D835Y and pHi in an isogenic Ba/F3 cell line model. Quizartinib, a type II FLT3 inhibitor, reduced pHi in Ba/F3 FLT3-ITD but not FLT3-ITD+D835Y cell lines. Crenolanib, a type I inhibitor that can inhibit FLT3 signal carrying ITD+D835Y mutation, and BRD7389 which inhibits the downstream signal p90-RSK, reduced pHi in both cell lines (Supplementary Fig. S3I). We have further examined the correlation between sensitivities of HMA, crenolanib, and BRD7389 in a larger cohort of CN primary AML carrying wildtype FLT3 or FLT3-ITD. FLT3-ITD AML were more sensitive to HMA, crenolanib, and BRD7389 (Supplementary Fig. S3J). Furthermore, the leukemia inhibitory effects of the 3 inhibitors were significantly correlated with each other supporting the proposition that the kinase cascade and NHE1 activity might act on the same axis in AML (Supplementary Fig. S3K).

### Combination treatment of kinase inhibitor and NHE1 inhibitor in AML

**Fig. 4:**
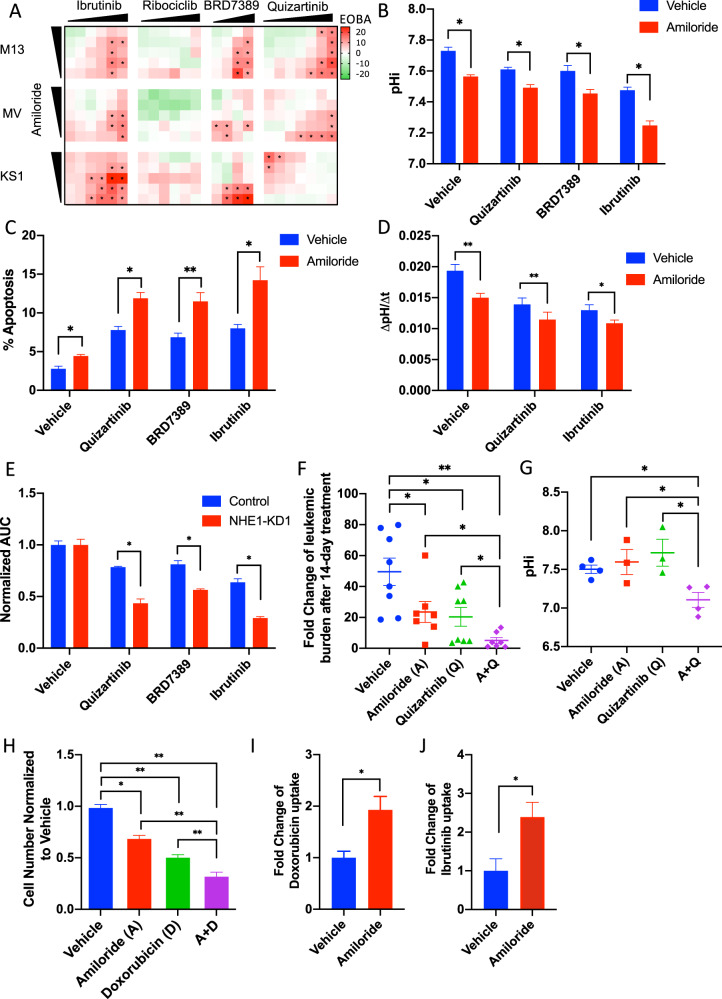
Combined kinase and NHE1 inhibition synergistically suppressed the growth of kinase-mutated AML. **A** Heatmap showing the Excess Over Bliss Additivism (EOBA) analysis of the growth inhibition from combined treatment with kinases inhibitors (ibrutinib 0.3–10 μM, ribociclib 0.3–10 μM, BRD7389 1–10 μM, and quizartinib 0.5–100 nM) and amiloride (0.6–10 μM) in MOLM-13, MV4-11, and Kasumi-1. EOBA over 10% were marked with an asterisk. **B**–**D** In vitro combined treatment with amiloride (10 μM) and kinase inhibitors (quizartinib 10 nM; BRD7389 10 μM; ibrutinib 10 μM) **B** reduced pHi (*n* = 4), **C** induced apoptosis (*n* = 4), and **D** suppressed NHE1 activity (*n* = 5) in MV4-11 compared to vehicle control. **E** NHE1-KD enhanced the growth inhibitory effect of kinase inhibitors in MV4-11 in vitro (*n* = 3). **F**, **G** Combined treatment with amiloride and quizartinib (A+Q) showed the greatest **F** in vivo growth inhibitory effect (*n* = 7–8) and **G** pHi reduction (*n* = 3–4) in MV4-11 engrafted in NSG mice, compared to those treated with vehicle control, amiloride or quizartinib only. **H** Amiloride (10 μM) enhanced the cytotoxic effect of doxorubicin in MV4-11 in vitro (*n* = 3). **I**, **J** Amiloride (10 μM) increased the intracellular distribution of **I** doxorubicin and **J** ibrutinib in MV4-11 in vitro (*n* = 5).

To ascertain the therapeutic potential of targeting NHE1 pathway in AML, the effects of FLT3, BTK, and CDK4 inhibition in combination with NHE1 inhibitor and KD were evaluated. p90-RSK inhibitor was included as a positive control. Quizartinib, ibrutinib but not ribociclib showed significant synergism with amiloride in suppressing cell growth in MOLM-13, MV4-11, and Kasumi-1 (Fig. 4A). Combination of kinase inhibitors and amiloride further induced apoptosis and reduced pHi of MV4-11 (Fig. 4B, C) and Kasumi-1 (Supplementary Fig. S4A, B). These effects were associated with increased inhibition of NHE1 activity upon combination treatment of amiloride and kinase inhibitor (Fig. 4D). Similar synergistic effects were seen with *NHE1*-KD (Fig. 4E and Supplementary Fig. S4C). In vivo, amiloride and quizartinib combination enhanced therapeutic effects of monotherapy in MV4-11 (Fig. 4F, G and Supplementary Fig. S4D, E) and Kasumi-1 (Supplementary Fig. S4F). Overexpression of NHE1 or MCT4, another pHi regulator [11], in MV4-11 attenuated the growth inhibitory effect of quizartinib and ibrutinib (Supplementary Fig. S4G, H), suggesting that kinase inhibition might depend on an acidic intracellular milieu.

### Potential synergism between amiloride and therapeutic agents

In addition to being an effective adjunct to tyrosine kinase inhibitors, amiloride also accentuated anti-leukemia effects of doxorubicin (Fig. 4H). We hypothesized that the potentiating effects of amiloride on anti-leukemic agents could be contributed by intracellular acidification, hence a reduced transcellular pH gradient and increased intracellular drug distribution. To prove the hypothesis, intracellular concentration of doxorubicin [29] and ibrutinib, both being weak bases, were measured in MV4-11 upon treatment with amiloride. Amiloride significantly increased doxorubicin and ibrutinib uptake inside cells (Fig. 4I, J). Collectively, the results indicated that increased intracellular concentration of therapeutic agents upon intracellular acidification by amiloride might explain the potentiating effects of the latter.

### Amiloride-containing serum from patients induced cell death in combination with kinase inhibitor

**Fig. 5:**
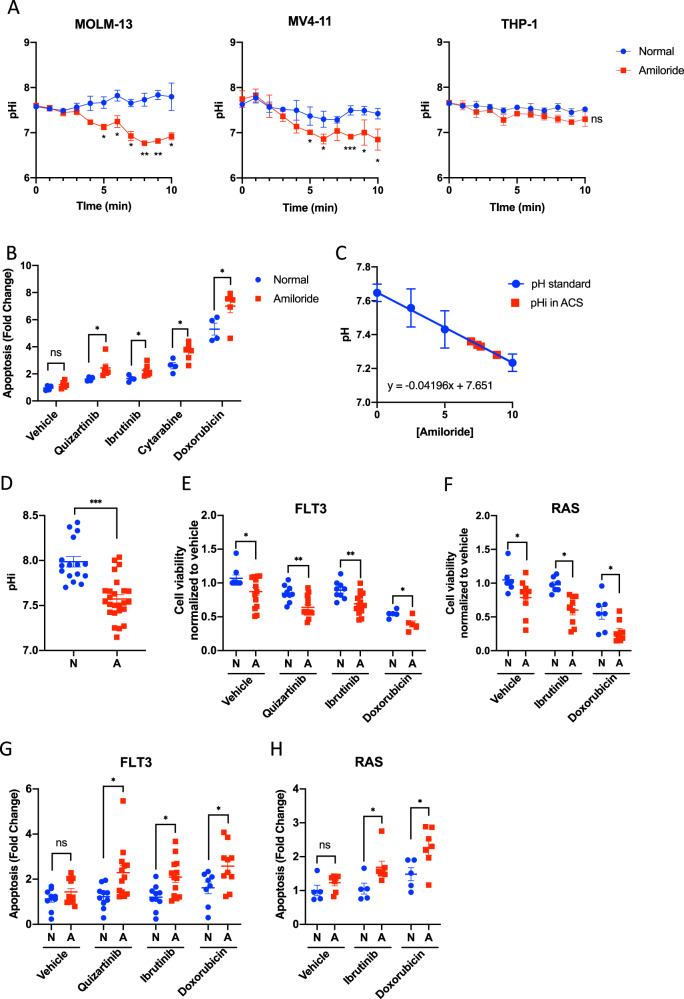
Amiloride-containing serum from patients was sufficient to acidify AML. **A** The pHi of MOLM-13 and MV4-11, but not THP-1, decreased in the culture of amiloride-containing serum (ACS) compared with the culture of serum from the normal donor in vitro (*n* = 3). **B** Culture of ACS enhanced the apoptosis induction of different therapeutic agents (quizartinib 10 nM, ibrutinib 10 μM, cytarabine 10 μM, and doxorubicin 100 nM) in MV4-11 compared with the culture of serum from the normal donor in vitro (*n* = 4–6). **C** Calibrated curve of pHi using MV4-11 against amiloride concentration. pHi was measured for this cell line cultured in ACS to estimate the effective amiloride concentration in serum (*n* = 4). **D** ACS significantly reduced pHi of primary AML with *FLT3* or *RAS* mutations (*n* = 16–25). **E**, **F** ACS reduced cell viability of primary AML samples carrying **E** *FLT3* (*n* = 5–14) or **F** *RAS* (*n* = 7–9) mutations and accentuated the leukemia inhibitory effects of quizartinib (10 μM), ibrutinib (10 μM), and doxorubicin (1 μM). **G**, **H** ACS accentuated the apoptotic effects of quizartinib, ibrutinib, and doxorubicin in primary AML samples carrying **G** *FLT3* (*n* = 8–13) and **H** *RAS* (*n* = 5–7) mutations. N: serum from healthy donors; A: amiloride-containing serum. In **D**–**H**, ACS from six individuals who were taking amiloride 20 mg daily for medical conditions unrelated to cancers were used. Serum from three healthy donors was used.

The observations aforementioned supported the proposition that inhibition of NHE1 by amiloride might be incorporated into the treatment algorithm for AML. We investigated if the serum level of amiloride, at the dose used for diuresis, was relevant for AML treatment. Serum from patients without blood diseases or cancers, who were given amiloride 20 mg for their underlying medical conditions, was obtained 4 hours after dosing. Serum from healthy donors was used as a control. The amiloride-containing serum (ACS) decreased pHi of MOLM-13 and MV4-11, but not that of THP-1, in a time-dependent manner (Fig. 5A). Furthermore, induction of apoptosis by kinase inhibition and chemotherapy in MOLM-13 was accentuated by ACS (Fig. 5B). We further examined the serum activity of amiloride in patients. A standard curve was constructed in which MOLM-13 was incubated in serum from normal donors with the addition of amiloride at defined concentrations and pHi was measured. A linear calibration was obtained (Fig. 5C). The cells were incubated in ACS and the changes in pHi were again measured. Effective amiloride concentration in ACS, based on the changes in pHi, was estimated to be 7.97 ± 0.95 µM (Fig. 5C), comparable with the effective concentration of amiloride used in in vitro study. The effects of ACS on primary AML samples were also examined. ACS significantly reduced pHi of primary AML with *FLT3* or *RAS* mutations (Fig. 5D). Moreover, ACS reduced cell viability of primary AML samples carrying *FLT3* (Fig. 5E, G) or *RAS* (Fig. 5F, H) mutations and accentuated the leukemia inhibitory and apoptotic effects of quizartinib, ibrutinib, and doxorubicin.

## Discussion

NHE1 activation and a resulting alkaline pHi have been associated with oncogenesis [7, 8]. However, its regulatory machinery, roles in leukemogenesis, and clinical relevance have not been addressed. We have previously demonstrated that Tescalcin (*TESC*) stabilized NHE1 to maintain an alkaline pHi and was associated with sorafenib resistance in *FLT3*-ITD AML [8]. In this study, we hypothesized that intracellular alkalization induced by NHE1 was crucial to the pathogenesis of other AML subtypes, and perturbation of this process might be considered for therapeutic intervention. We identified 13 kinases whose expression led to NHE1 activation in normal hematopoietic progenitors, including FLT3-ITD, KRAS^G12D^, and BTK. These kinases induced phosphorylation of NHE1, raised pHi, and increased cell growth in vitro. NHE1 activities and phosphorylation as well as sensitivities to NHE1 inhibitor and knockdown were more pronounced in primary samples and AML cell lines with gain-of-function kinase mutations. Kinase inhibitors including quizartinib and ibrutinib reduced NHE1 phosphorylation and pHi in kinase-mutated AML cell lines and such responses were ameliorated by NHE1 mutations at sites critical for its phosphorylation, suggesting a direct mechanistic link between kinase activation, NHE1 phosphorylation, and activation. Intriguingly, ribociclib reduced pHi but not NHE1 phosphorylation, suggesting yet identified effects on pHi that was NHE1 independent. It is presently unclear if interruption of cell cycle and hence proliferation by ribociclib might cause intracellular acidification. If so, the anti-leukemia effects of ribociclib and amiloride might be distinct and their combined effects could be additive, which was consistent with our observations. Therapeutically, amiloride enhanced the anti-leukemic effects of kinase inhibitors and chemotherapy, at least partially by increasing their intracellular uptake. The results were clinically relevant, as the plasma NHE1 inhibitory activities in kinase-mutated AML cells could be readily demonstrated in patients taking amiloride for diuresis. These observations were consistent with the reported roles of NHE1 activation in oncogenesis. On the other hand, there were a number of novel findings with particular reference to AML that were of clinical and therapeutic relevance.

First, intracellular alkalization via NHE1 activation was of pathogenetic significance in AML with kinase activation including *FLT3*, *RAS,* and *KIT* mutations. In vitro and in vivo studies showed that AML cell lines and primary AML samples carrying these mutations were addicted to NHE1 activation as evidenced by higher level of NHE1 phosphorylation and basal activities as well as increased sensitivities to NHE1 inhibition and knockdown. The proliferative effects of mutated kinases were potentiated by NHE1 expression in normal hematopoietic cells. Direct binding of kinases to NHE1 could be demonstrated and specific mutations of NHE1 abrogated the effects of kinase inhibitors, highlighting the pathogenetic significance of NHE1 phosphorylation. Our findings underscored the significance of NHE1 activation and intracellular alkalization in kinase-mutated AML, which were associated with unsatisfactory response to conventional chemotherapy and inferior patient outcome [5, 30–32].

Second, we demonstrated a hitherto undescribed role of *BTK* in AML pathogenesis. Overexpression of *BTK* in normal hematopoietic progenitors induced NHE1 phosphorylation and activation, resulting in an increase in pHi and cellular proliferation. In AML cell lines carrying *FLT3*-ITD and *KIT* mutations, ibrutinib, a BTK inhibitor, reduced NHE1 phosphorylation and lowered pHi. Ibrutinib not only inhibited BTK phosphorylation, but also that of ERK and p90-RSK, both of which have been shown to activate NHE1 via phosphorylation [18]. In fact, specific mutation of NHE1 at sites critical for phosphorylation by AKT, p90-RSK, and ERK abrogated the effects of ibrutinib. Early studies have shown constitutive activation of BTK in AML [33] and its role in AML-niche interaction in BM via SDF1/CXCR4 axis [34] or interaction with toll-like receptor 9 (TLR9) [35]. BTK inhibition has also been shown to sensitize AML toward venetoclax and chemotherapy [36, 37]. In this study, we further demonstrated a novel role of BTK in supporting intracellular alkalization and conferring proliferative advantage to AML.

Third, we demonstrated that combined inhibition of NHE1 and its activating kinases synergistically suppressed leukemia growth, associated with a decrease in pHi and induction of apoptosis. Synergistic effects were also seen upon combination treatment with amiloride and chemotherapy, suggesting that additional mechanisms may be involved in the therapeutic effects of NHE1 inhibition. In fact, amiloride treatment substantially increased intracellular uptake of chemotherapy and ibrutinib, for which such assays were available. A decrease in pHi induced by amiloride could shift the transmembrane H^+^ equilibrium and favor intracellular distribution of weak bases like doxorubicin [38] and ibrutinib. Similar phenomena have been shown for sorafenib [8].

**Fig. 6:**
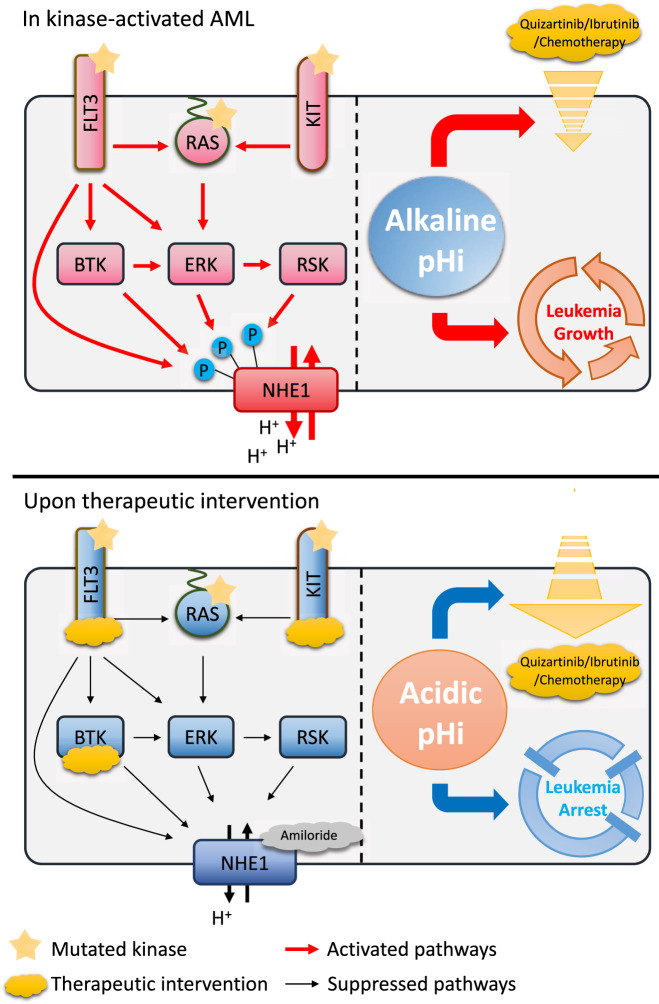
Kinase-dependent activation of NHE1 in AML and overcoming aberrant pHi by therapeutic means. In kinase-activated AML, NHE1 was phosphorylated and activated by kinase cascade and pHi became alkaline. The latter enhanced leukemia growth and reduced the intracellular distribution of therapeutic agents such as kinase inhibitors and chemotherapy. NHE1 could be inhibited by amiloride or kinase inhibitors that inhibited its upstream activators. These treatments reduced pHi and hence reversed the pathogenetic processes of leukemia and increased intracellular distribution of therapeutic agents.

These observations supported a pathogenetic model in kinase-mutated AML. Gain-of-function mutations and overexpression of kinases including FLT3-ITD, KRAS^G12D^, KIT^N822K^, and BTK, provide proliferative signals to AML (Fig. 6) by promoting cell cycle and inhibiting autophagy transcriptionally and post-translationally [39–43]. We demonstrated a novel kinase-regulated proliferative signal, via phosphorylation of NHE1 directly, or indirectly via their downstream effectors ERK and p90-RSK at specific sites, leading to its activation hence a rise in pHi and promotion of leukemic survival [11]. These pathologic processes were perturbed by inhibition of kinase and NHE1. The resulting intracellular acidification also favored intracellular distribution of kinase inhibitors and chemotherapeutic agents, accentuating their anti-leukemia effects. Regulation of NHE1 was tissue specific and compared with other cancer tissues, AML cell lines were less dependent on calmodulin and ROCK for survival [online cell line database Depmap (https://depmap.org/portal/)]. It might explain their lack of effects on leukemia growth and pHi in the present study, even at maximum dose of 10 μM for both calmodulin (CGS9343B: IC50 = 3.3 nM) and ROCK-RhoA inhibitors (HA1100: IC50 = 0.73 μM).

Our results were of clinical relevance. For decades, amiloride has been used as diuretics via NHE1 inhibition in the renal tubules. It was unknown if plasma concentration of amiloride achievable in patients would be relevant to its anti-leukemia effects. We demonstrated that the plasma amiloride level was sufficient in inhibiting NHE1 in AML cells, leading to intracellular acidification and apoptosis in combination with tyrosine kinase inhibitors and chemotherapy. On the other hand, normal hematopoietic progenitors were unaffected by NHE1 inhibition [8]. The observations may provide a foundation for future clinical trials based on NHE1 inhibitor as an adjunctive therapy for kinase-mutated AML.

## Materials and methods

### Cell processing

Mononuclear cells from blood and/or BM of AML patients were purified using Ficoll-Paque^TM^ Plus (Amersham Biosciences, Uppsala, Sweden) and stored in liquid nitrogen until use. Patients and donors gave informed consent, and the procurement of these materials was approved by the institution review board in accordance with the Declaration of Helsinki. Leukemia cell lines included in this study were summarized in Supplementary Table S1.

### In vitro culture of primary AML cells and cord blood

Primary AML cells and CB CD34^+^ progenitors were cultured in SFEMII (StemCell Technologies, Canada) with 1% P/S, recombinant IL-3 (20 ng/ml), IL-6 (20 ng/ml), SCF (100 ng/ml), FLT3-L (100 ng/ml) and G-CSF (20 ng/ml) (PeproTech, NJ, USA). Leukemia cell lines were cultured *as per* standard protocols. DMSO (0.1%) was used as vehicle control. Cell growth after drug treatment was enumerated by PrestoBlue^TM^ Cell Viability Reagent (ThermoFisher Scientific, MA, USA).

### Measurement of pHi and NHE1 activity assay

pHi was measured by SNARF-1 in cell lines (ThermoFisher Scientific), pHrodo^TM^ Red in primary AML samples (ThermoFisher Scientific), or pH reporter mCherry-SEpHluorin as reported previously [8, 11]. In brief, PBS-washed AML cells were incubated with SNARF-1 or pHrodo^TM^ Red for 37 °C, washed and re-suspended in PBS. The fluorescence intensities (SNARF-1: ratio of 580 and 640 nm; pHrodo^TM^ Red: 580 nm) were measured by CytoFLEX Flow Cytometer (Beckman Coulter, CA, USA). NHE1 activity was analyzed as reported previously [44]. After incubation in SNARF-1, AML cells were treated with drugs for 4 hours and then acid loaded with 20 mM NH_4_Cl for 5 min followed by Na^+^ free Krebs’ modified buffer solution [140 mM tetramethylammonium chloride, 5.4 mM KCl, 2.8 mM CaCl_2_, 1.2 mM MgSO_4_, 0.3 mM NaH_2_PO_4_, 10 mM HEPES, 5 mM glucose, pH 7.4] for 15 min. Thereafter, the cells were replenished with Na^+^ (140 mM). pHi recovery was measured for 10 min at 30-s intervals by CLARIOstar microplates reader (BMG Labtech, NC, USA).

### Kinase library screening

Myristoylated Kinase Library (Addgene #1000000012) was used in this study. In HEK293FT, the kinase-containing plasmids were transfected into mCherry-SEpHluorin stably expressing HEK293FT cells using Lipofectamine 2000 (ThermoFisher Scientific) according to the manufacturer’s protocol. pHi was measured by CLARIOstar microplates reader on day 2 post transfection. In CB, the kinase was packaged as retro-virus and infected into CB CD34^+^ progenitor cells by spinoculation (800 g for 2 hours at 32 °C). Geneticin (1.5 mg/ml) was added to infected CB cells to select positively infected cells for 7 days at day 3 post infection. pHi was then determined by SNARF-1.

### Immunoprecipitation

Cells were harvested and lyzed using NP-40-based lysis buffer with protease and phosphatase inhibitors. Total protein (500 μg) in 1 ml lysate was incubated with 2 μg of anti-NHE1 antibody (Abcam, UK) or anti-FLT3 antibody (Santa Cruz Biotechnology, TX, USA) [45] at 4 °C overnight. The antibody-protein complex was pulled down using Protein G sepharose (ThermoFisher Scientific) at 4 °C for 2 hours. The sepharose was washed twice with lysis buffer and eluted by SDS protein loading dye by boiling for 5 min. The supernatant was collected and stored at −80 °C for further analysis.

### Western blot analysis

Cell lines or primary AML samples were lyzed in CelLytic^TM^ MT Cell Lysis Reagent (Sigma-Aldrich, MO, USA) containing protease and phosphatase inhibitors. Cell lysate was separated, transferred, and blotted with primary (Supplementary Table S2) and horseradish peroxidase (HRP)-conjugated secondary antibodies. HRP signal was examined and amplified by chemiluminescent substrate such as Amersham ECL western blot detection reagents (GE Healthcare, UK) or Luminata Forte Western HRP substrate (Millipore, MA, USA) and evaluated by the ChemiDocTM XRS+ System (Bio-Rad, CA, USA).

### Lentiviral induction

HEK293FT cells were plated into a T175 flask and co-transfected with CMVD9 (21 μg), PUDG (7 μg), and lentiviral plasmids (21 μg) (pLVTHM-GFP-Scrambled shRNA or pLVTHM-GFP-NHE1 shRNA) (Supplementary Table S3) by Lipofectamine 2000 (ThermoFisher Scientific). pLVTHM was a gift from Didier Trono (Addgene plasmid # 12247) [46]. Virus containing medium was collected after 2 and 3 days post transfection, filtered, and ultra-centrifuged at 37,000 rpm (Beckman Coulter) at 4 °C for 2 hours. The supernatant was discarded and the virus pellet was re-suspended in 200 μl OPTI-MEM^®^I medium (ThermoFisher Scientific), aliquoted in 20 μl and stored in −80 °C.

Scrambled sequence or NHE1 shRNA was transduced into AML cell lines (1 × 10^6^) by spinoculation using a 24-well plate that contained 1 ml culture medium per well RPMI-1640 (ThermoFisher Scientific) together with viral supernatant and polybrene (8 μg/mL, Sigma-Aldrich). Thereafter, the cells were washed and re-suspended in 1 ml medium (RPMI-1640). Successful transduction was determined by GFP^+^ expression and the cells were sorted by MoFlo^®^ XDP High-Speed Cell Sorter (Beckman Coulter).

Leukemic alleles (MLL-AF9, FLT3-ITD, and KRAS^G12D^) or kinases (BTK and CDK4) were transduced retro-virally into CB CD34^+^ progenitor cells by spinoculation. Positively infected cells were selected by geneticin (1.5 mg/ml) for 7 days.

### Cell proliferation assay

Cell proliferation rate was also determined by CellTrace^TM^ Violet Cell Proliferation Kit (ThermoFisher Scientific) according to the manufacturer's protocol. In brief, on day 1 post drug treatment in AML, cells were stained with the dye and cultured for 2 more days. The fluorescence intensity of CellTrace^TM^ Violet was then examined by FACS.

### Colony-forming assay

Clonogenic activity of leukemia cells was evaluated by methylcellulose-based culture (MethoCult^TM^ H4230, StemCell Technologies). MV4-11 was seeded at 100 cells/ml in triplicates and colonies were examined after 10 days of culture.

### Xenotransplantation and in vivo drug treatment

GFP-shRNA transduced or luciferase-GFP expressing leukemia cell lines were injected intravenously into sublethally irradiated (200 cGy) 6–8-week-old female NSG mice. At week 4 post transplantation, human leukemia engraftment was evaluated in luciferase-expressing leukemic cell line, mice were injected with 150 mg/kg D-luciferin (Promega, WI, USA) intraperitoneally and bioluminescence was examined using IVIS Spectrum in vivo imaging system (Perkin-Elmer, MA, USA). Engrafted mice were randomized, unblinded, and treated with amiloride (Sigma-Aldrich, 20 mg/kg every 2 days, i.p. injection) or quizartinib (LC lab, USA, 1.2 mg/kg daily, i.p. injection) and bioluminescence was reassessed on day 14 of treatment. At humane endpoint or time of death, the mouse BM was harvested for FACS analysis to demonstrate engraftment by human AML cells (AML-GFP^+^/mouse-CD45.2^-^/PI^-^) and for in vitro culture to demonstrate their viability. The studies were approved by the Committee of the Use of Laboratory Animals for Teaching and Research (CUTALR) of the University of Hong Kong.

### Doxorubicin and ibrutinib uptake assay

MV4-11 was primed with amiloride (10 μM) for 30 min and subjected to doxorubicin and ibrutinib uptake assay. For doxorubicin, amiloride-treated MV4-11 was cultured with doxorubicin (1 μM) for 1 hour and washed with PBS twice. The fluorescent intensity of intracellular doxorubicin was measured by CytoFLEX Flow Cytometer (Ex: 561 nm, Em: 585 nm). For ibrutinib, amiloride-treated MV4-11 was cultured with ibrutinib (1 μM) for 1 hour. The cell was then washed with PBS twice and cultured with PCI-33380 (10 μM), a fluorescent probe of BTK inhibitor. Cells were then washed with PBS twice and the fluorescent intensity of intracellular PCI-33380 was measured by CytoFLEX Flow Cytometer (Ex: 488 nm, Em: 525 nm).

### Statistics

Results were expressed as mean ± standard error of the mean. Groups of data were compared by Mann–Whitney *U*-test for numerical data and *χ*^2^ for categorical data. Level of correlation was obtained using Pearson R. Synergism of drug combination was evaluated by Excess over Bliss Additivism. Mouse survival after AML cell line engraftment was evaluated by Kaplan–Meier analysis. All experiments have been replicated at least three times (two-tailed test, *p < 0.05; **p < 0.01; ***p < 0.001).

## Supplementary information

Legend to supplementary figures

Supplementary Figure 1

Supplementary Figure 2

Supplementary Figure 3

Supplementary Figure 4

Supplementary Table
