# Supplementary material for: Regulation of proton partitioning in kinase-activating acute myeloid leukemia and its therapeutic implication
Source: Leukemia. 2022 May 27;36(8):1990–2001. doi: 10.1038/s41375-022-01606-0 (PMC9343251; doi:10.1038/s41375-022-01606-0)
Supplement: Supplementary file 1 — Legend to supplementary figures [file 41375_2022_1606_MOESM1_ESM.pdf]

Legend to Supplemental Figure S1: (A) Representative Western blot analysis of phosphoserine 14-3-3 (pSer 14-3-3) and NHE1 in NHE1 IP eluate in amiloride-sensitive (Kasumi-1, MOLM-13 and MV4-11) and resistant (THP-1, OCI-AML3 and KG-1) AML cell lines. (B-E) Amiloride (10 μM) and HMA (10 μM) treatment significantly (B) suppressed growth (n=3), (C) reduced pHi (n=3), (D) induced apoptosis (n=4) and (E) suppressed proliferation (n=4) of Kasumi-1, MOLM-13 and MV4-11, but not other human AML cell lines. (F) Representative Western blot analysis of phosphoserine 14-3-3 (pSer 14-3-3) and NHE1 in NHE1 IP eluate in primary AML samples carrying either *FLT3*, *RAS* or *KIT* mutation controlled by AML samples without *FLT3/RAS/KIT* mutation. (G) Primary AML samples carrying different mutations showed no significant difference in cellular viability measured by Prestoblue^{TM} after 3-day *in vitro* culture (n=29).

Legend to Supplemental Figure S2: (A) *NHE1* expression was successfully knocked down by shRNA in THP-1 and OCI-AML3 examined by quantitative RT-PCR analysis (n=3). (B-C) *In vitro NHE1* knockdown did not affect (B) pHi or (C) growth of THP-1 and OCI-AML3 (n=3). (D) *NHE1* expression was successfully knocked down by shRNA in primary AML samples carrying *FLT3* and *RAS* mutation examined by quantitative RT-PCR analysis (n=12).

Legend to Supplemental Figure S3: In (A-B) MV4-11 and (C-D) Kasumi-1, treatment with kinase inhibitors significantly reduced (A, C) the level of NHE1 phosphorylation and (B, D) pHi *in vitro* (n=3). (E-F) Only quizartinib (10 nM) and BRD7389 (10 μM), but not CGS 9343B (10 μM) and HA1100 (10 μM), (E) suppressed the growth and (F) reduced pHi of Kasumi-1, MOLM-13 and MV4-11 *in vitro* (n=3). (G) Effect of overexpression of BTK, FLT3-ITD or CDK4 and treatment with ibrutinib (10 μM), quizartinib (10 nM), ravoxertinib (100 nM) or ribociclib (10 μM) on pHi of HEK293 with wildtype and mutated *NHE1* (n=3). (H) Representative Western blot analysis of FLT3, BTK, ERK and NHE1 in NHE1/FLT3 IP in MV4-11. (I) Treatment with crenolanib and BRD7389, but not quizartinib, reduced the pHi of Ba/F3 carrying *FLT3-ITD* and *FLT3-ITD+D835Y* mutation *in vitro* (n=3). (J) Primary AML samples carrying *FLT3* mutation were more sensitive towards *in vitro* treatment with HMA (10 μM), crenolanib (10 μM) and BRD7389 (10 μM), compared to AML with wildtype *FLT3* (n=32-55). (K) AUC of crenolanib, HMA and BRD7389 significantly correlated with each other in primary AML samples (n=87).

Legend to Supplemental Figure S4: (A-B) Treatment of amiloride (10 μM) enhanced (A) pHi acidification and (B) apoptosis induction in combination with kinase inhibitors (quizartinib 100 nM; BRD7389 10 μM; ibrutinib 10 μM) in Kasumi-1 *in vitro* (n=3). (C) NHE1-KD enhanced the growth inhibitory effect of kinase inhibitors in Kasumi-1 *in vitro* (n=3). (D) Leukemic burden analysis by bioluminescence in MV4-11 engrafting NSG mice upon *in vivo*

amiloride and quizartinib treatment (n=7-8). (E) Survival analysis of NSG mice engrafted with MV4-11 and MOLM-13 upon combined *in vivo* treatment with amiloride and quizartinib showed longer survival compared to those treated with vehicle control, amiloride or quizartinib only (n=10-12). The grey dash line indicated the start date of treatment. (F) Combined treatment with amiloride and quizartinib showed the greatest *in vivo* growth inhibitory effect in Kasumi-1 engrafted in NSG mice, compared to those treated with vehicle control, amiloride or quizartinib only (n=4-5). (G-H) The growth inhibition upon the treatment of amiloride (10 μM), quizartinib (10 nM) and ibrutinib (10 μM) were diminished in the presence of (G) NHE1 or (H) MCT4 overexpression *in vitro* (n=3).
