# Supplementary figures and images for: Regulation of proton partitioning in kinase-activating acute myeloid leukemia and its therapeutic implication
Source: Leukemia. 2022 May 27;36(8):1990–2001. doi: 10.1038/s41375-022-01606-0 (PMC9343251; doi:10.1038/s41375-022-01606-0)

# Supplementary Figure S1

A

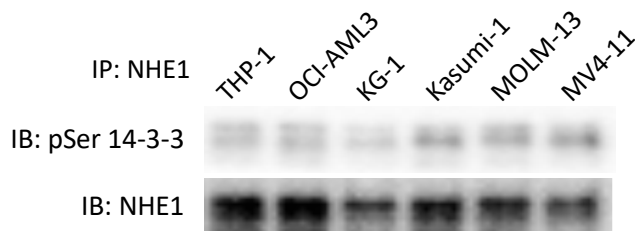

B

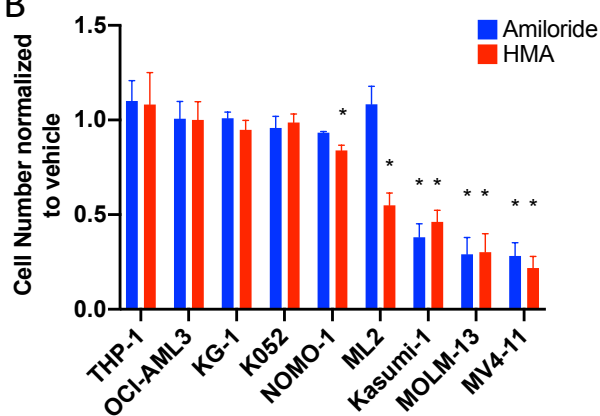

C

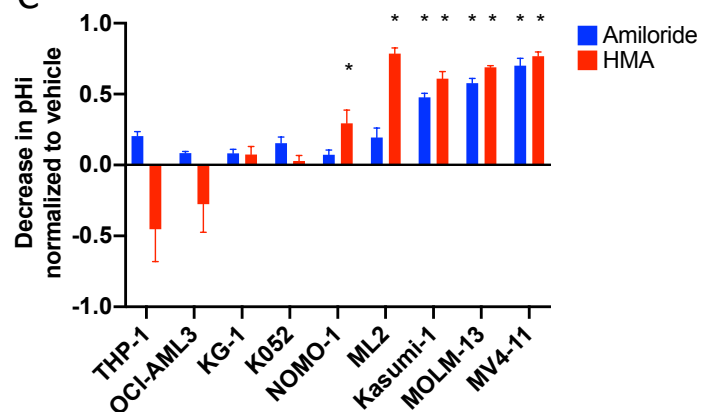

D

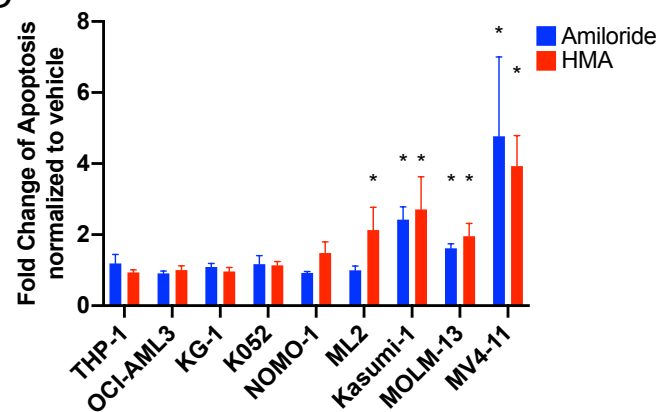

E

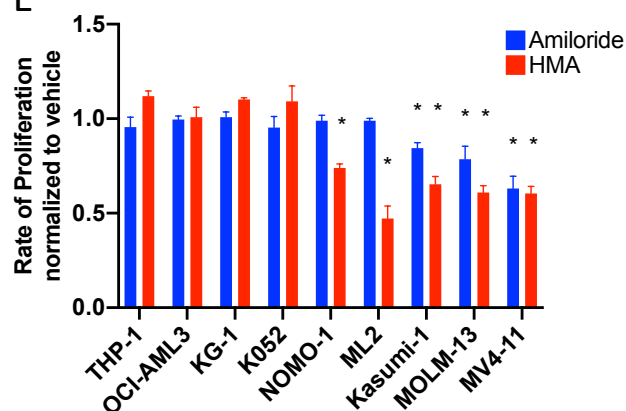

G

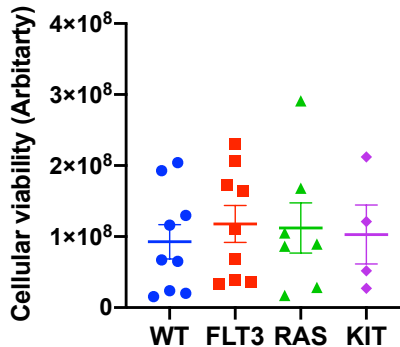

F

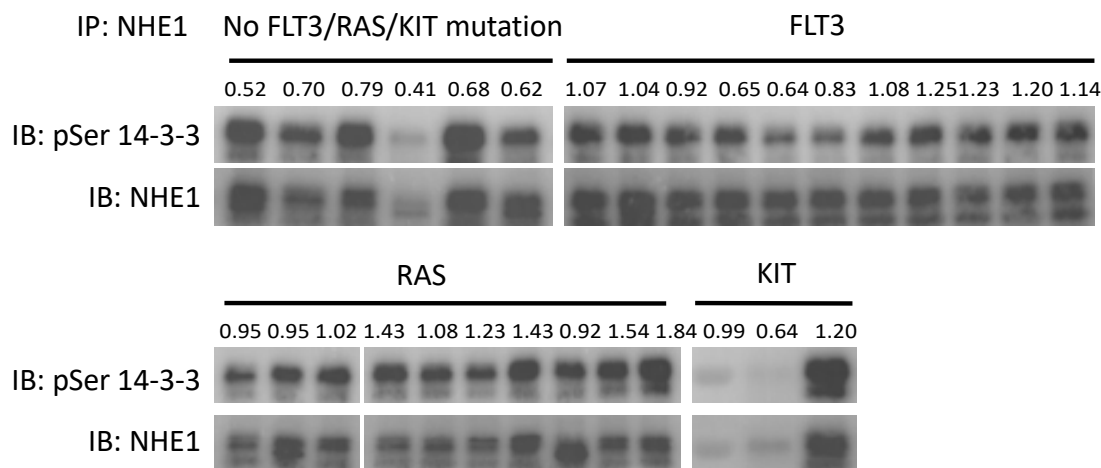

Supplement: Supplementary file 2 — Supplementary Figure 1 [file 41375_2022_1606_MOESM2_ESM.pdf]

Supplementary Figure S2

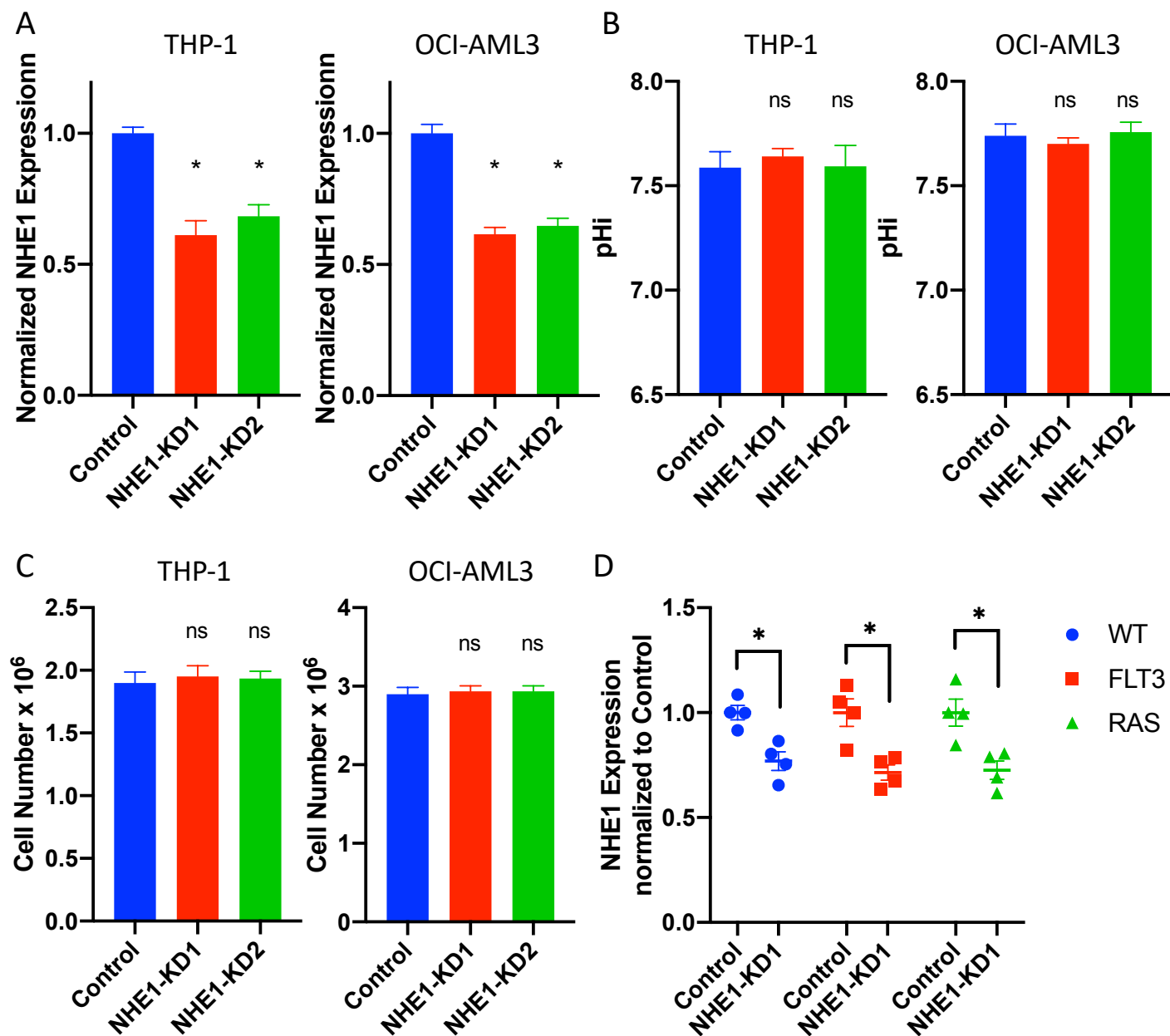

Supplement: Supplementary file 3 — Supplementary Figure 2 [file 41375_2022_1606_MOESM3_ESM.pdf]

Supplementary Figure S3

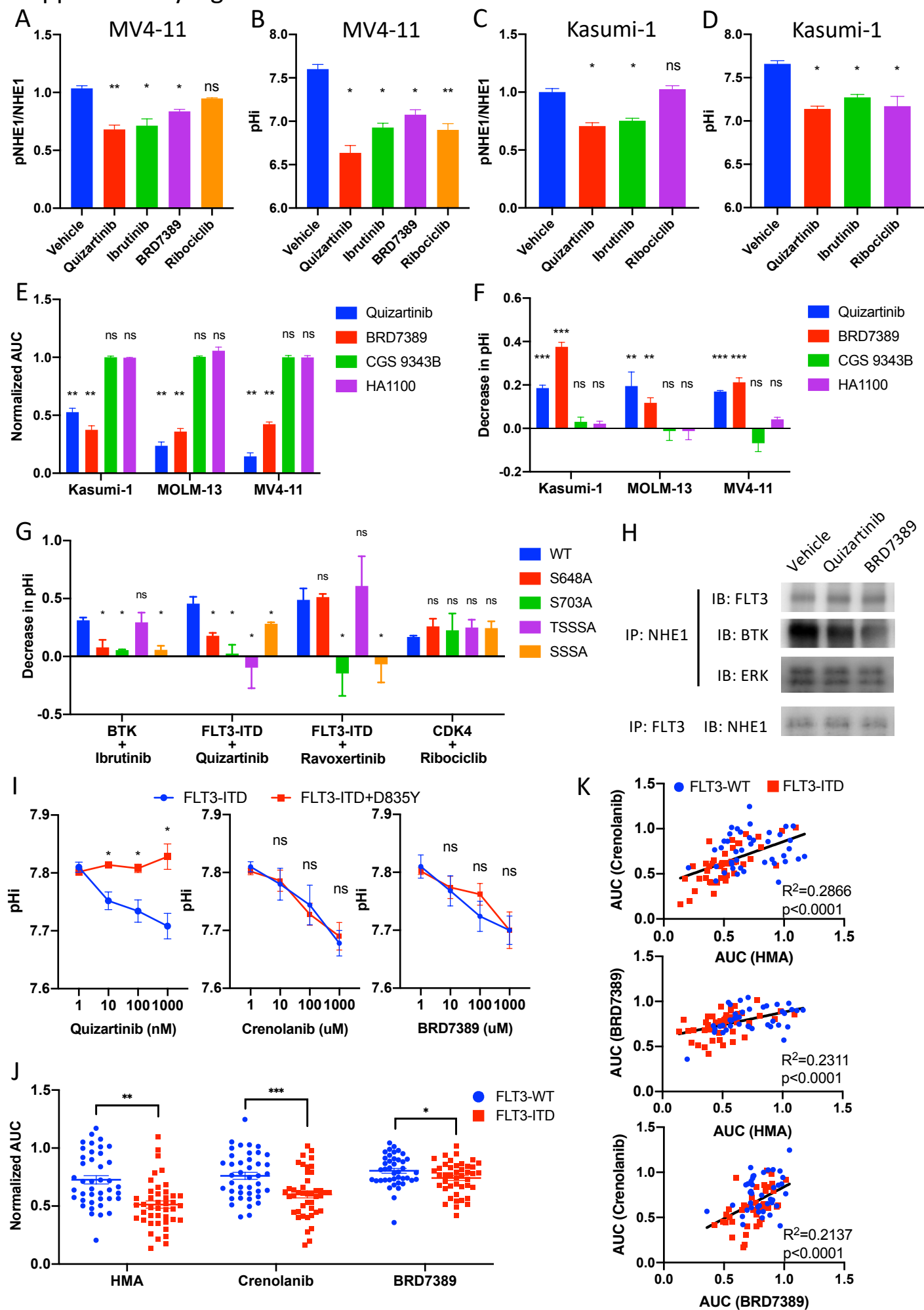

Supplement: Supplementary file 4 — Supplementary Figure 3 [file 41375_2022_1606_MOESM4_ESM.pdf]

Supplementary Figure S4

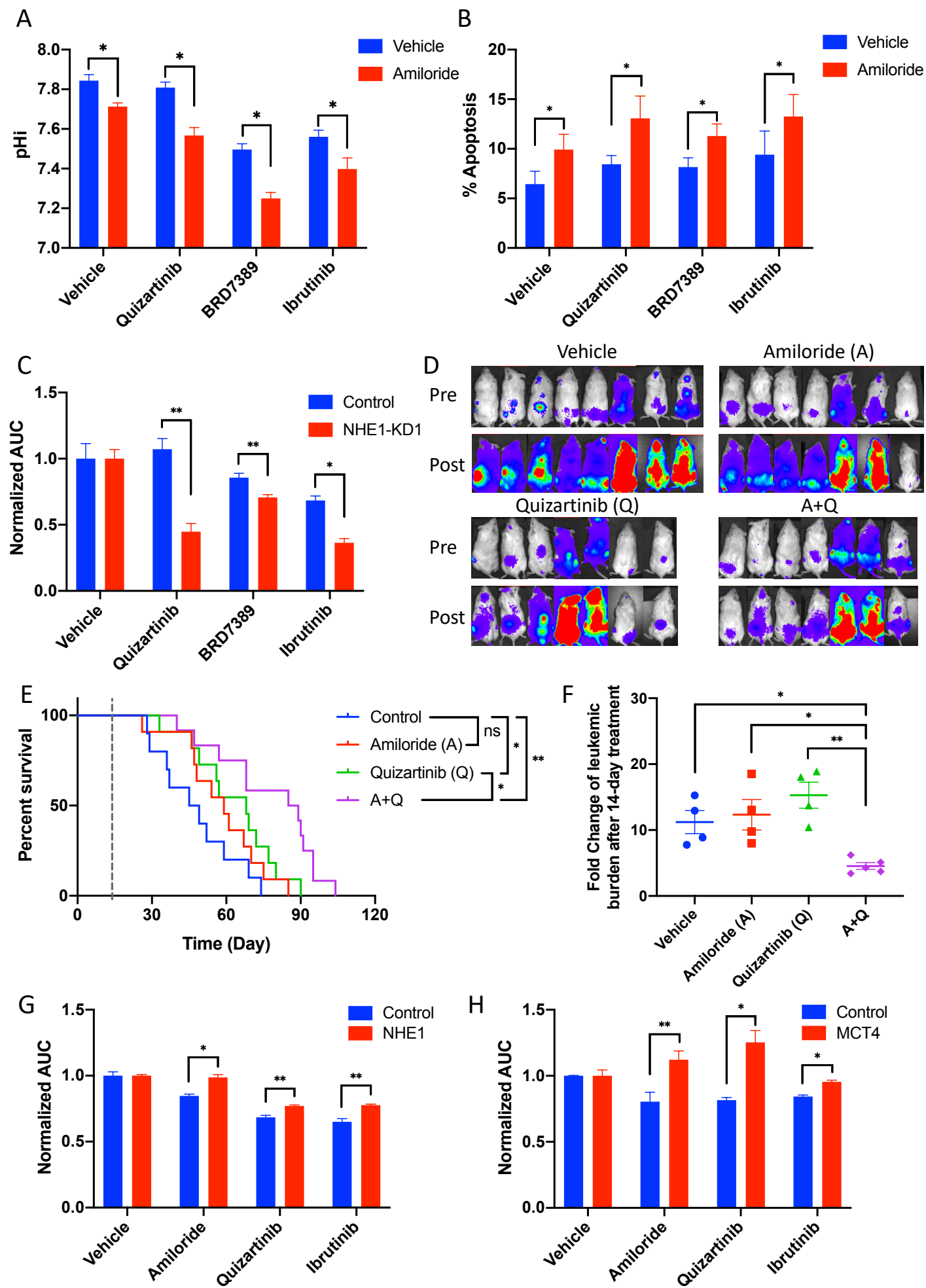

Supplement: Supplementary file 5 — Supplementary Figure 4 [file 41375_2022_1606_MOESM5_ESM.pdf]
