# Supplementary material for: Regulation of proton partitioning in kinase-activating acute myeloid leukemia and its therapeutic implication
Source: Leukemia. 2022 May 27;36(8):1990–2001. doi: 10.1038/s41375-022-01606-0 (PMC9343251; doi:10.1038/s41375-022-01606-0)
Supplement: Supplementary file 6 — Supplementary Table [file 41375_2022_1606_MOESM6_ESM.pdf]

Supplemental Table S1

Genetics information of human leukemic cell lines

Cell line	Cell type	Genetics
THP-1	AML	MLL-AF9, CSNK2A1-DDX39B, $NRAS^{G12D}$, $TP53^{del}$
OCI-AML3	AML	$DNMT3A^{R882C}$, $NPM1^{typeA}$
KG1	AML	FGFR10P2-FGFR1, $NRAS^{G12D}$
K052	AML	$DNMT3A^{R882H}$, $NRAS^{G13R}$, $TP53^{R248W}$
NOMO-1	AML	MLL-AF9, $KRAS^{G13D}$, $TP53^{del}$
ML2	AML	MLL-AF6, $KRAS^{A146T}$
Kasumi-1	AML	AML1-ETO, KIT^{N822L}, $TP53^{R248Q}$
MOLM-13	AML	MLL-AF9, FLT3-ITD
MV4-11	AML	MLL-AF4, FLT3-ITD
U937	AML	CALM-AF10, $PTEN^{del}$, $TP53^{del}$
K562	CML	BCR-ABL, $TP53^{del}$
Jurkat	T-ALL	$NOTCH1^{R1627H}$, $TP53^{del}$, BAX^{del}
KE-37	T-ALL	$NOTCH1^{del}$, $NRAS^{G12D}$, $RB1^{G310E}$, $TP53^{del}$
PEER	T-ALL	$NOTCH1^{L1600P}$, NUP214-ABL1, $TP53^{W163C}$
KMS-12-PE	MM	$TP53^{R337L}$
RPMI-8226	MM	IGKV2-28-IGKJ4, $EGFR^{T751I}$, $KRAS^{G12A}$, $TP53^{E285L}$, $TRAF3^{del}$

Supplementary Table S2

List of antibodies

Target	Source	Dilution	Manufacturer
FLT3	Rabbit	1:200	Santa Cruz Biotechnology
p-FLT3	Rabbit	1:1000	Cell Signaling Technology
BTK	Rabbit	1:1000	Cell Signaling Technology
p-BTK	Rabbit	1:1000	Cell Signaling Technology
ERK	Rabbit	1:1000	Cell Signaling Technology
p-ERK	Rabbit	1:1000	Cell Signaling Technology
RSK1	Rabbit	1:1000	Cell Signaling Technology
p-RSK1	Rabbit	1:1000	Cell Signaling Technology
NHE-1	Rabbit	1:250	Abcam
p-Ser 14-3-3 binding motif	Rabbit	1:1000	Cell Signaling Technology
β-actin	Mouse	1:10000	Sigma Aldrich

Supplementary Table S3

Sequences of NHE1 shRNAs

siRNA1 sense

5’-CGCGCAAGCTCAACCGGTTTAATAACTCGAGTTATTAAACCGGTTGAGCTTGTTTTT-3’

siRNA1 anti-sense

5’-CGAAAAACAAGCTCAACCGGTTTAATAACTCGAGTTATTAAACCGGTTGAGCTTG-3’

siRNA2 sense

5’-CGCGCAGACCAATCTTAGTTTCTAACTCGAGTTAGAAACTAAGATTGGTCTGTTTTT-3’

siRNA2 anti-sense

5’-CGAAAAACAGACCAATCTTAGTTTCTAACTCGAGTTAGAAACTAAGATTGGTCTG-3’
